# Sources of variation in Affymetrix microarray experiments

**DOI:** 10.1186/1471-2105-6-214

**Published:** 2005-08-29

**Authors:** Stanislav O Zakharkin, Kyoungmi Kim, Tapan Mehta, Lang Chen, Stephen Barnes, Katherine E Scheirer, Rudolph S Parrish, David B Allison, Grier P Page

**Affiliations:** 1 Section on Statistical Genetics, University of Alabama at Birmingham, Birmingham, Alabama, USA; 2 Departments of Pharmacology and Toxicology, University of Alabama at Birmingham, Birmingham, Alabama, USA; 3 Heflin Center for Human Genetics, University of Alabama at Birmingham, Birmingham, Alabama, USA; 4 Department of Bioinformatics and Biostatistics, University of Louisville, Louisville, Kentucky, USA

## Abstract

**Background:**

A typical microarray experiment has many sources of variation which can be attributed to biological and technical causes. Identifying sources of variation and assessing their magnitude, among other factors, are important for optimal experimental design. The objectives of this study were: (1) to estimate relative magnitudes of different sources of variation and (2) to evaluate agreement between biological and technical replicates.

**Results:**

We performed a microarray experiment using a total of 24 Affymetrix GeneChip^® ^arrays. The study included 4^th ^mammary gland samples from eight 21-day-old *Sprague Dawley CD *female rats exposed to genistein (soy isoflavone). RNA samples from each rat were split to assess variation arising at labeling and hybridization steps. A general linear model was used to estimate variance components. Pearson correlations were computed to evaluate agreement between technical and biological replicates.

**Conclusion:**

The greatest source of variation was biological variation, followed by residual error, and finally variation due to labeling when *.cel files were processed with dChip and RMA image processing algorithms. When MAS 5.0 or GCRMA-EB were used, the greatest source of variation was residual error, followed by biology and labeling. Correlations between technical replicates were consistently higher than between biological replicates.

## Background

Microarray chips are a powerful technology capable of measuring expression levels of thousands of genes simultaneously. Expression profiling has led to dramatic advances in the understanding of cellular processes at the molecular level, which may lead to improvements in molecular diagnostics and personalized medicine [1]. The number of experiments involving microarrays grows nearly exponentially each year [2]. Several platforms are currently available, including the commonly used short oligonucleotide-based Affymetrix GeneChip^® ^arrays, which utilize multiple probes for each gene and automated control of the experimental process from hybridization to quantification. Although microarrays have tremendous potential, great effort and care is required in planning and designing microarray experiments, analyzing gene expression data, and interpreting results [3-6].

A typical microarray experiment has many different sources of variation which can be attributed to biological and technical causes [4]. Biological variation results from tissue heterogeneity, genetic polymorphism, and changes in mRNA levels within cells and among individuals due to sex, age, race, genotype-environment interactions and other factors [7-10]. Biological variation reflects true variation among experimental units (i.e. individual mice, rats, tissue samples, etc.) and is of interest to investigators. However, preparation of samples, labeling, hybridization, and other steps of microarray experiment can contribute to technical variation, which can significantly impact the quality of array data [11-16]. To ensure highly reproducible microarray data, technical variation should be minimized by controlling the quality of the RNA samples, and by efficient labeling and hybridization [17].

Identifying sources of experimental variation and assessing their magnitude are important for optimal experimental design, as for example, in the planning of mRNA pooling in microarray experiments [18]. Similarly, this information is useful for estimating the optimal number of required technical replicates because measurement accuracy and reliability affect researchers' power to identify differentially expressed genes [19]. However, other considerations, such as the goals of the study, the features of a particular microarray platform, or the cost of arrays and samples may influence experimental design [4-6]. Several studies have been conducted to examine the relative contributions of various factors in different experimental settings [7-15]. Here, we estimated the relative magnitudes of sources of variation in experiments involving Affymetrix GeneChip^® ^arrays and evaluated agreement between biological and technical replicates.

## Results

### Experimental design

**Figure 1:**
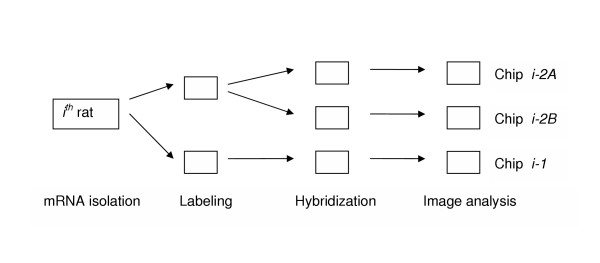
**Experimental design. **The scheme of hierarchical unbalanced design used in our experiment is shown. A total of 8 rats and 24 chips were used.

The experiment was set up as described in Materials and Methods (see Figure 1). Source *.cel data files from 24 GeneChip^® ^arrays were subjected to image processing by four popular methods for probe-level data implemented in BioConductor [20]: DNA Chip Analyzer (dChip) [21], MAS 5.0 [22], RMA [23], and GCRMA-EB [24].

### Variance components estimation

**Figure 2:**
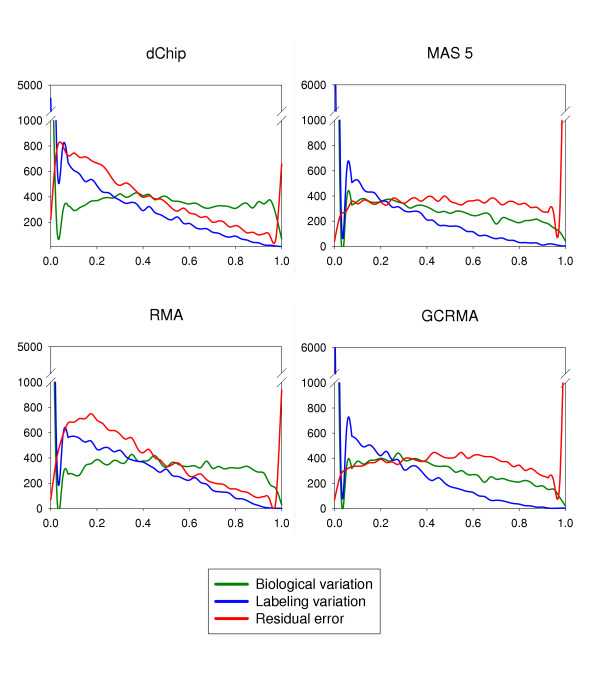
**Density plots of different sources of variation. **Density plots of relative magnitudes of different sources of variation are shown for data analyzed with four image processing algorithms. The proportions of different variance components are shown on x-axis and frequencies of probe sets are shown on y-axis.

For each probe set, expression levels were modeled as follows: *y_g _*= *μ_g _*+ *B_g _*+ *L*(*B*)_*g *_+ *ε_g_*, where *B_g _*~ *N*(0, ) is the effect of biological variation among experimental units; *L*(*B*)_g _~ *N*(0, ) is the effect of labeling nested within biological replications; and, *ε_g _*~ *N*(0, ) is the residual error. It should be noted that in our case biological variation could be confounded by technical variation arising during tissue isolation and preparation of mRNA samples. Dobbin et al., 2005, found that variation at this stage of microarray processing was small compared to variation at the hybridization step [25]. The model was fit separately on the gene expression measurements of each of dChip, MAS 5.0, RMA and GCRMA-EB probe set summaries. Both the effects of biological replication and the labeling effect nested within biological cases were treated as random. We estimated variance components and applied shrinkage variance estimators to them. These shrunken estimators borrow information across genes and have been shown to improve statistical tests [26]. Figure 2 shows the density plots of the distributions of relative magnitudes of different sources of variation. The results indicated that for most of the genes, the biggest source of variation was biological when using dChip and RMA, whereas the biggest source of variation was residual error when using GCRMA-EB or MAS 5.0. For all algorithms, a significant number of probe sets had biological and labeling variance components estimates equal or very close to zero. The findings are summarized in Table 1.

**Table 1:** Proportions of different sources of variation

Source	dChip		MAS 5.0		RMA		GCRMA-EB	
	Mean	SD	Mean	SD	Mean	SD	Mean	SD
Biological variation	0.431	0.304	0.292	0.300	0.393	0.306	0.310	0.292
Labeling variation	0.206	0.230	0.136	0.198	0.221	0.224	0.147	0.192
Residual error	0.363	0.274	0.572	0.311	0.386	0.272	0.543	0.298

### Assessment of reproducibility

**Figure 3:**
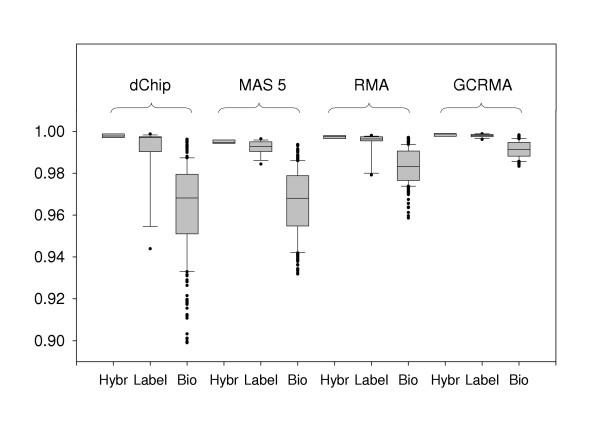
**Boxplots of pairwise correlations between chips. **Box plots of Pearson correlations between technical replicates at the hybridization step (Hybr; *i*_*2A *vs. *i*_*2B *chips, where *i *is biological replicate), labeling step (Label; *i*_*1 *vs. *i*_*2A *and *i*_*1 *vs *i*_*2B *chips), and between different biological replicates (Bio; all pairwise combinations) are shown for four image processing algorithms (dChip, MAS 5.0, RMA, GCRMA-EB). Technical replicates have consistently higher correlations than different biological replicates.

**Figure 4:**
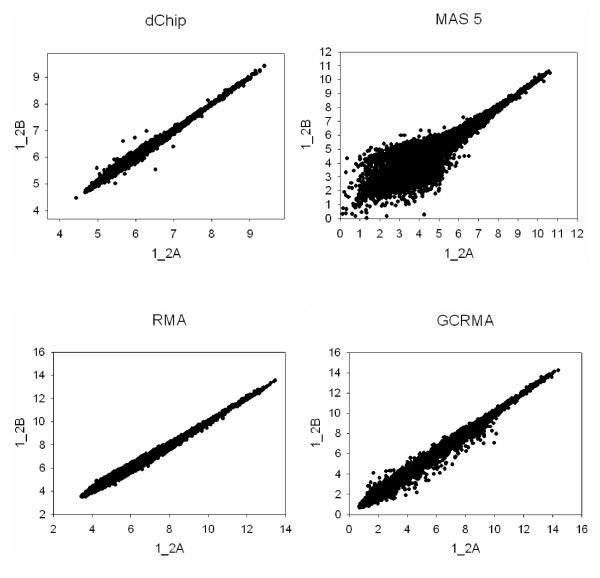
**Comparison of two technical replicates of the same biological replicate using different image processing techniques. **Expression levels detected on the *1*_*2A *chip (x-axis) are plotted against levels detected on the *1*_*2B *chip (y-axis). Results obtained with different image processing algorithms are shown. dChip and MAS 5.0 are shown on the log scale for compatibility with RMA and GCRMA-EB. Good agreement between two chips will result in data grouped along the identity line, while lack of agreement will lead to dispersion.

**Figure 5:**
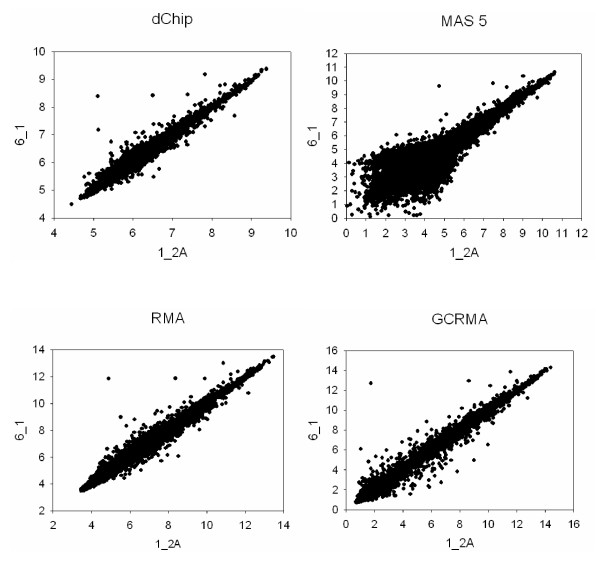
**Comparison of two different biological replicates using different image processing techniques. **Expression levels detected on the *1*_*2A *chip (x-axis) are plotted against levels detected on the *6*_*1 *chip (B) (y-axis). Results obtained with different image processing algorithms are shown. dChip and MAS 5.0 are shown on the log scale for compatibility with RMA and GCRMA-EB. Good agreement between two chips will result in data grouped along the identity line, while lack of agreement will lead to dispersion.

We investigated agreement between technical replicates and biological replicates using Pearson correlations between chips. The correlations for the following three groups were compared: (1) Correlations between two technical replicates at the hybridization stage within a biological replicate (i.e., chips *i*_*2A *vs. *i*_*2B*; total of 8 correlations); (2) Correlations between two technical replicates at the labeling stage within a biological replicate (i.e. chips *i*_*1 *vs. *i*_*2A *and *i*_*1 *vs. *i*_*2B*; total of 16 correlations); (3) Correlations between different biological replicates (all possible pairwise comparisons; total of 252 correlations). Results indicated that technical replicates at the hybridization step agree more closely (i.e. have consistently higher correlations) either than technical replicates at the labeling stage or than different biological replicates (Figure 3). This finding can be illustrated using scatter plots: regardless of the image processing method, technical replicates of the same biological replicate (Figure 4) show less dispersion than data from different animals (Figure 5).

The reproducibility at the hybridization stage was assessed by testing the significance of the differences between expression levels of technical replicates at the hybridization step using a paired t-test analysis as described in Material and Methods. Briefly, for each probe set we tested the hypothesis that a difference in expression levels between two technical replicates (i.e., between *i*_*2A *and *i*_*2B *chips) is equal to zero. A total of 15,923 paired t-tests were conducted and 15,923 p-values obtained for each image processing algorithm. The distribution of p-values was modeled using a mixture model approach [27]. Under a global null hypothesis, there are no differentially expressed genes and distribution of p-values is expected to be uniform on [0, 1]. If some genes are truly differentially expressed, we expect an increased number of small p-values (near 0). Distributions of p-values for the data obtained by four image processing methods are presented on Figure 5. By fitting the mixture of two beta distributions, one can estimate proportion of differentially expressed genes. We obtained the following estimates: dChip – 10.8%; MAS 5.0 – 4.8%; RMA – 2.3%, and GCRMA-EB – 13.6%. Thus, at the nominal α-level 0.05, the number of differentially expressed genes was smaller than expected by chance when data were processed with MAS 5.0 or RMA, but above the nominal α-level when data was processed with dChip or GCRMA-EB.

## Discussion

Using Affymetrix GeneArray^® ^chips, we examined the relative magnitudes of different sources of variation in microarray experiment. Analysis of variance using mixed-effects linear models is a common way to account for and test the significance of various factors contributing to overall variation [3]. Due to limitations of our hierarchical unbalanced experimental design and relatively small number of degrees of freedom, we did not include factors that can potentially contribute to variation such as day of processing, scanning order, mRNA preparation, etc. We assume that such factors were not significant. However, to formally test this assumption, another experiment is needed.

We used a general linear model to partition variance for each probe set into three components. The first source was biological (i.e. animal-to-animal) variation. The biological variation may be confounded by technical variation at the mRNA preparation step, but this variation is probably relatively small compared to variation at the hybridization step [25]. Thus, we assume that most of the variation for this effect was due to true biological differences among animals. The second source of variation was the effect of labeling. Although our experiments were carried out by the same person, using the same equipment, under the same experimental conditions as much as realistically possible, there is always some variation caused by minor environmental differences in temperature, duration, pipetting etc., which influences labeling efficiency. The third source of variation other than animal-to-animal variation and labeling-effect variation was residual error caused by differences in hybridization, scanning and other factors. To compare the relative magnitudes of different sources of variation, we estimated variance components and applied shrunken variance estimators that borrow information across genes. We constructed these shrunken variance estimators by shrinking a group of individual variance estimators toward their common corrected geometric mean [26]. The amount of shrinkage depends on the variation on the individual variance components estimators. These estimators were shown to be robust in respect to variance heterogeneity in gene expression data among groups [26].

We found that our results depend on the image processing algorithm used: biological variation was the largest source when dChip or RMA were used, but when *.cel files were processed with GCRMA-EB or MAS 5.0, the largest source was residual error. Bakay et al., 2002, found that biological variation presumably caused by tissue heterogeneity and genetic polymorphism was a major source of variation while technical variation was minor [12]. Han et al., 2004, found that biological variation was about of the same size as other sources combined [14]. Whitney et al., 2003, found that inter-individual variation in gene expression profiles was correlated with gender, age, and the time of day at which the sample was taken. These intrinsic differences in expression patterns were likely caused by differences in genotype, although they might also reflect epigenetic or environmental factors [9]. Oleksiak et al., 2002, in their studies of teleost fish have observed significant differences in gene expression levels between individuals from the same population and between different populations. These differences could be caused by genetic variation as well as other factors, including maternal effects and genotype-environment interactions [10]. On the contrary, Dumur et al., 2004, found that day-to day variation was the main source of variation [17]. Woo et al., 2004, in studies of inbred mice strains, detected that most of the genes had small biological variance, but about 10% of genes showed large variation between individuals [28].

We found that technical replicates within a biological replicate had higher and more consistent correlations with each other than with other biological replicates. Generally, our correlations were higher than those observed by Dobbin et al., 2005, for interlaboratory correlations between tumor samples [25] and were compatible with values for in-lab correlations obtained in another study [29].

The consistency of the hybridization step was evaluated using paired t-tests following by modeling of distribution of resulting p-values. The significance depends on the image processing algorithm used: the hybridization effect was not significant for MAS 5.0 (4.8% of genes were differentially expressed between two technical replicates) and RMA (2.3% of genes), but the proportion of differentially expressed genes was higher than expected by chance for dChip (10.8% of genes) and GCRMA-EB (13.6% of genes).

The low-level data were analyzed using four popular methods implemented in the BioConductor [20] package: dChip [21], MAS 5.0 [22], RMA [23], and GCRMA-EB [24]. We found that different low-level data processing algorithms produced different results. We provide comparisons mainly to illustrate the compatibility of several algorithms. Evaluation of the strengths and weaknesses of different image processing algorithms may require other experimental settings, such as spike-in data. Shedden et al., 2005, performed a comprehensive comparison of seven image processing methods for Affymetrix arrays and demonstrated that the choice of image processing algorithm has a major impact on the results of microarray data analysis [30]. The authors found that the dChip method operates consistently well, while MAS 5.0 and GCRMA-EB consistently performed poorly. GCRMA-EB had a particular disagreement with other methods when a t-test was used for group comparison, presumably because it might be more sensitive to the underlying statistical assumptions of a test (e.g. independence of genes). Similarly, we observed that estimates of the proportion of differentially expressed genes between two technical replicates at the hybridization stage were different than those for data processed with GCRMA-EB compared to other methods, which is consistent with finding of Shedden et al. [30].

The results presented here are specific for the systems being studied, and other experimental conditions may yield different estimates. For example, we used an outbred strain of rats, which had greater inherent biological variation than inbred strains. In cell cultures of inbred mice strains under otherwise equal conditions, the relative magnitude of biological variation presumably would be smaller. Different steps in microarray data analysis, such as normalization, transformation, and gene filtering, may affect results as well [31-35]. A microarray platform and microarray facility can also have a significant impact, as was demonstrated in several recent studies [25,36-38]. Testing the influence of these various factors could be an interesting topic of future research.

## Conclusion

Identification of sources of variation and their relative magnitudes, among other factors, is important for optimal experimental design and the development of quality control procedures. In this study, we evaluated the relative magnitudes of different sources of variation in Affymetrix microarray experiments. Different image processing algorithms gave different variance components estimates: the greatest source was animal-to-animal (i.e. biological) variation when dChip and RMA were used, and residual error when MAS 5.0 or GCRMA-EB were used. We observed that correlations between technical replicates within one biological replicate were consistently higher than between different biological replicates. It should be noted that estimates obtained here were specific for our experimental system, and results would probably change if we used another organism or tissue, or another microarray platform.

## Methods

### Samples and microarrays

This study included samples taken from eight 21-day-old *Sprague Dawley CD *female rats exposed to genistein (a soy isoflavone) via their mother's milk. The mothers were fed AIN-76A diet supplemented with 200 mg genistein / kg chow. Young rats were sacrificed at day 21 and the 4^th ^mammary glands extracted and flash-frozen in liquid nitrogen within 3 minutes of ex-sanguination. Samples were frozen at -70°C for approximately 90 days, at which point the extraneous fat was dissected off and samples processed in Trizol RNA extraction buffer. Total RNA was generated using Affymetrix RNA extraction and labeling kits according to manufacturer's protocols, and each of the RNA samples was split in half. The first half was labeled and run on a RAE 230A Affymetrix GeneChip^®^, and the other half was labeled, split, and run across two RAE 230A chips (see Figure 1). Affymetrix arrays were run in the Genomics Core facility of the Heflin Center for Human Genetics at the University of Alabama at Birmingham. Images were scanned on a HP 2500 scanner.

### Image processing

Each of the low-level *.cel data files was processed using four popular image analysis algorithms: DNA Chip Analyzer (dChip) [21], MAS 5.0 [22], RMA [23], and GCRMA-EB [24]. The processing was done in R 1.8.1 / R 1.9.1 [39]. The default settings for all normalization procedures were used as implemented in the BioConductor [20]; in particular, the scale normalization for MAS 5.0; the quantile-quantile normalization for RMA; the invariant-set normalization for dChip; and the quantile-quantile normalization for GCRMA-EB (see [35] for the details of the different normalization methods). The default implementation of dChip, RMA, and GCRMA-EB used only the PM (perfect match) intensity matrix, while MAS 5.0 by default used both PM and MM (mismatch) matrices.

### Evaluation of relative magnitudes of different sources of variation

The relative magnitudes of different sources of variation were estimated using a general linear model in PROC VARCOMP procedure of SAS 9.1 (SAS Institute Inc., Cary, NC) using REML option. The expression levels of each probe set, *y_g_*, were modeled as follows: *y_g _*= *μ_g _*+ *B_g _*+ *L*(*B*)_*g *_+ *ε_g_*, where *B_g _*~ *N*(0, ) is the effect of biological variation among experimental units; *L*(*B*)_*g *_~ *N*(0, ) is the effect of labeling variation nested within biological replications; and *ε_g _*~ *N*(0, ) is the residual error, i.e. technical variation caused by factors other than labeling. Biological effect could be confounded by technical variation arising during mRNA sample preparation. For each probe set, variance components were estimated. We applied shrinkage variance estimators that borrow information across probe sets and improve individual variance estimators by shrinking them toward their corrected geometric mean [26]. The total variance was assumed to be the sum of three components: *VAR_Tot _*= *VAR_Bio _*+ *VAR_Label _*+ *VAR_Residual_*, where *VAR_Bio _*is the shrunken estimate of biological variance; *VAR_Label _*is the shrunken estimate of variance due to labeling; and *VAR_Residual _*is the shrunken variance estimate of residual error. The relative proportion of each source of variation was calculated as a ratio of the shrunken variance estimate to the sum of all three shrunken variance estimates:, i.e.  calculates the proportion of biological variation,  calculates the proportion of variation due to labeling within biological replicates, and  calculates the proportion of variation due to unaccounted technical variation (residual error).

### Assessment of reproducibility across different replicates

Pearson correlations between chips were calculated for the following three groups: (1) Correlations between two technical replicates at the hybridization step (i.e., chips *i*_*2A *vs. *i*_*2B*; total of 8 correlations); (2) Correlations between two technical replicates at the labeling step (i.e. chips *i*_*1 *vs. *i*_*2A *and *i*_*1 *vs. *i*_*2B*; total of 16 correlations); (3) Correlations between different biological replicates (all possible pairwise comparisons; total of 252 correlations).

To evaluate the significance of variation introduced at the hybridization step, paired t-tests were performed on 16 chips (*i*_*2A *and *i*_*2B *chips from each of 8 separate rats). For each probe set, the null hypothesis was that the difference between the expression levels of two replicates was equal to zero. A total of 15,923 t-tests were performed and 15,923 p-values were generated for each image processing algorithm. The distribution of resulting p-values was modeled using a mixture of two beta distributions [24]. If the global null hypothesis is true, there are no differentially expressed genes and the distribution of p-values is expected to be uniform [0, 1]. We expect an increased number of p-values close to 0 if some genes are truly differentially expressed. By fitting the mixture of two beta distributions, one can estimate a proportion of differentially expressed genes. At the nominal α-level 0.05, one expects 5% of genes to be differentially expressed just by chance. Thus, the differences between replicates were considered significant only if the proportion of differentially expressed genes was > 5%.

## Authors' contributions

SOZ performed analysis of the data, drafted and finalized the manuscript. KK and RP helped with analysis and contributed to discussion. KES performed microarray experiment that generated the data. SB provided support for microarray experiment and contributed to discussion. TM and LC analyzed the data with four image-analysis algorithms. GPP planned and designed the experiment. GPP and DBA supervised and coordinated the project and assisted with the interpretation. All authors have read and approved the manuscript.

**Figure 6:**
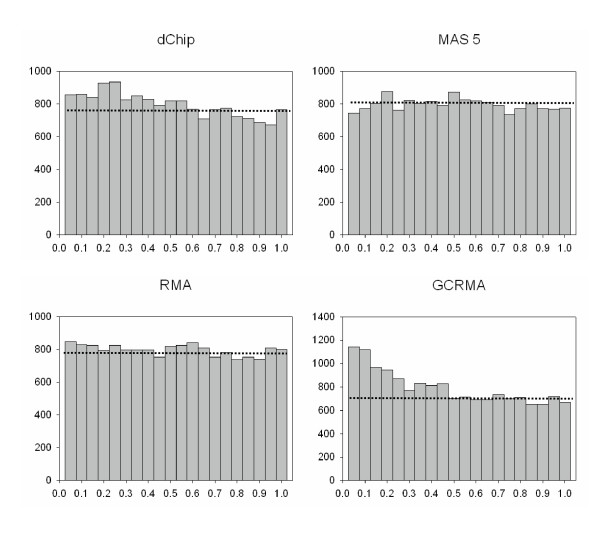
**Distributions of p-values for the paired t-test for hybridization effect. **Histograms of p-values for four image processing algorithms. If the global null hypothesis is true, the distribution of p-values would be uniform from 0 to 1 (dotted line). If differentially expressed genes are present, the number of small p-values will be increased.
